# Recent Progress in Biosensors for Environmental Monitoring: A Review

**DOI:** 10.3390/s17122918

**Published:** 2017-12-15

**Authors:** Celine I. L. Justino, Armando C. Duarte, Teresa A. P. Rocha-Santos

**Affiliations:** 1 Department of Chemistry & CESAM, University of Aveiro, Campus de Santiago, 3810-193 Aveiro, Portugal; aduarte@ua.pt (A.C.D.); ter.alex@ua.pt (T.A.P.R.-S.); 2 ISEIT/Viseu, Instituto Piaget, Estrada do Alto do Gaio, Galifonge, Lordosa, 3515-776 Viseu, Portugal

**Keywords:** antibodies, aptamers, biosensors, environmental monitoring, enzymes, pesticides, pollutants

## Abstract

The environmental monitoring has been one of the priorities at the European and global scale due to the close relationship between the environmental pollution and the human health/socioeconomic development. In this field, the biosensors have been widely employed as cost-effective, fast, in situ, and real-time analytical techniques. The need of portable, rapid, and smart biosensing devices explains the recent development of biosensors with new transduction materials, obtained from nanotechnology, and for multiplexed pollutant detection, involving multidisciplinary experts. This review article provides an update on recent progress in biosensors for the monitoring of air, water, and soil pollutants in real conditions such as pesticides, potentially toxic elements, and small organic molecules including toxins and endocrine disrupting chemicals.

## 1. Introduction

Biosensors can be classified according to their transduction principle such as optical (including optical fibre and surface plasmon resonance biosensors), electrochemical (including amperometric, and impedance biosensors), and piezoelectric (including quartz crystal microbalance biosensors) or based on their recognition element as immunosensors, aptasensors, genosensors, and enzymatic biosensors, when antibodies, aptamers, nucleic acids, and enzymes are, respectively, used. In environmental monitoring, the majority of biosensors are identified as immunosensors and enzymatic biosensors, but recently the development of aptasensors has been increased, due to the advantageous characteristics of aptamers such as easiness to modify, thermal stability, in vitro synthesis, and possibility to design their structure, to distinguish targets with different functional groups, and to rehybridize [1].

The research on the construction of biosensors for environmental monitoring of organic pollutants, potentially toxic elements, and pathogens has been contributed to the sustainable development of society due to the problems of environmental pollution for human health. Traditional analytical methods employed for the environmental monitoring of pollutants include various chromatographic techniques (for example, gas chromatography and high performance liquid chromatography coupled with capillary electrophoresis or mass spectrometry), but they require expensive reagents, time-consuming sample pre-treatment, and expensive equipment [2,3]. Thus, more sensitive, cost-effective, rapid, easy to operate, and portable biosensing devices are urgently needed to monitor such pollutants responsible for adverse effects on ecosystems and human health, overcoming the magnification of environmental problems. For example, traditional methods are not effective for in situ measurements as in the case of accidental release of pesticides or acute poisoning, where rapid, miniaturized, and portable equipment is needed such as environmental monitoring biosensors [4,5,6]. In this direction, the role of nanotechnology on the development of fast and smart biosensing devices is crucial to the success of the detection of environmental pollutants; the majority of recent biosensors includes nanomaterials and novel nanocomposites in their system, advantageous for the improvement of analytical performance such as sensitivity and limit of detection [7]. For example, gold nanostructures could be a promising and versatile platform for enzyme immobilization matrix due to their high surface area and good electron mediation capability, stabilizing the enzyme through electrostatic interactions [2,8]. In addition, gold nanoparticles displayed excellent biocompatibility and little citotoxicity even in vivo [8]. Thus, the intrinsic contribution of nanotechnology and biotechnology could improve the analytical performance of sensing systems in order to develop more commercial environmental monitoring biosensors for in situ measurements, which effectively constitutes a current technical and expectant challenge in the environmental field. The main barrier in the commercialization of such biosensors is related to the interdisciplinary context of fabrication and limitations on the in situ operation and on the analytical performance, mainly in reproducibility. In addition, commercially available environmental biosensors are also limited to their application in real samples since the majority of biosensors is successfully tested in buffered solutions or distilled water contaminated by environmental pollutants and, when applied to real samples, the matrix effect influenced their analytical performance. In the environmental context, few commercial biosensors are still available with the main application on biochemical oxygen demand biosensing [9]. For pollutant detection, the company MicroVacuum Ltd. (Budapest, Hungary) is specialized in the commercialization of optical label-free biosensors, and, for environmental applications, a label-free immunosensor for the herbicide trifluralin is available using optical waveguide lightmode spectroscopy [10]. In the literature, only oldest works employed adapted commercially available biosensors for environmental monitoring, such as in the case of optical immunosensors based on surface plasmon resonance principle for 2,4-dichlorophenol [11] and dichlorodiphenyltrichloroethane (DDT) [12] detection from DKK-TOA Corporation (Tokyo, Japan) and SENSIA S.L. (Gipuzkoa, Spain), respectively.

This article provides an overview on the recent applications of biosensors for environmental monitoring over the last five years (2013–2017) applied for the detection of pesticides such as organophosphorous pesticides, pathogens such as bacteria, potentially toxic elements such as Hg^2+^, and small organic molecules including toxins and endocrine disrupting chemicals. Future perspectives on the field of environmental monitoring with biosensors were also provided.

## 2. Biosensors for Environmental Monitoring

Biosensors including immunosensors, aptasensors, genosensors, and enzymatic biosensors have been reported for the detection and monitoring of various environmental pollutants, using antibodies, aptamers, nucleic acids, and enzymes as recognition elements, respectively. Table 1 provides a summary of recent biosensors for environmental monitoring, whose applications and analytical performances are discussed and compared in this section.

### 2.1. Pesticides

Due to their significant presence in the environment, the pesticides are among the most important environmental pollutants. For example, the organophosphorous insecticides are extensively used in agriculture and constitute a group of pesticides with major environmental concern due to their high toxicity. Thus, simple, sensitive, and miniaturized in situ methodologies such as biosensors have been developed as analytical strategies for their detection and monitoring without the need of extensive sample pre-treatment, as described in the following.

#### 2.1.1. Organophosphorous Pesticides

For the detection of organophosphorous insecticides using paraoxon as the model analyte, disposable amperometric enzymatic (acetylcholinesterase) biosensors were proposed using a cysteamine self-assembled monolayer on gold screen-printed electrodes [4]. The disposable biosensors showed a linear range up to 40 ppb with a limit of detection of 2 ppb and sensitivity of 113 µA mM cm^−2^. The good analytical performance could be attributed to the highly oriented enzyme immobilization using the self-assembled monolayer. After tested in river water samples spiked with 10 ppb of paraoxon, recoveries of 97 ± 5% (*n* = 3) were obtained, demonstrating the effectiveness of such enzymatic biosensors. In addition, the use of disposable screen-printed electrodes dispenses of time-consuming procedures such as reactivation of the immobilized enzymes using, for example, obidoxime solution and pralidoxime iodide (PAM) or the use of renewable enzymatic membrane that would be needed for a second application of the biosensors [4]. The same research group proposed another electrochemical biosensor for paraoxon detection in wastewater samples [13] but using butyrylcholinesterase instead of acetylcholinesterase and carbon black nanoparticles instead of a cysteamine self-assembled monolayer in the transduction element [4]. The use of carbon black nanoparticles is advantageous due to the low applied potential, ease of preparing a stable dispersion for mass-produced sensing systems, and cost-effectiveness [13]. In addition, the carbon black nanoparticles allowed the formation of a superficial uniform film after enzyme immobilization. Recently, other biosensors such as amperometric acetylcholinesterase biosensor based on gold nanorods [2] and colorimetric biosensor based on the iodine-starch colour reaction and multi-enzymes (acetylcholinesterase and choline oxidase) [5] were reported for the detection of paraoxon in real water samples. Limits of detection of 4.7 ppb (17 nM) and 0.7 nM were obtained with the colorimetric biosensor and the amperometric biosensor, respectively, which were lower than the maximum residue level in the European Union pesticides database (10 ppb) [73]. In addition, the colorimetric biosensor was applied for the detection of paraoxon in vegetable irrigation water with recoveries of 88–110%, and the amperometric biosensor was applied in river water and seawater samples with recoveries of 96–98%, demonstrating their reliability for paraoxon detection. In the case of the amperometric biosensor, a reactivation step was employed in order to recover the activity of immobilized acetylcholinesterase. The immobilized enzyme was reactivated after pesticide determination by immersion in a solution containing nucleophilic compounds cholinesterase reactivator (PAM, 5.0 mM solution) and 95% of its original current was restored, demonstrating the good reactivation.

**Figure 1:**
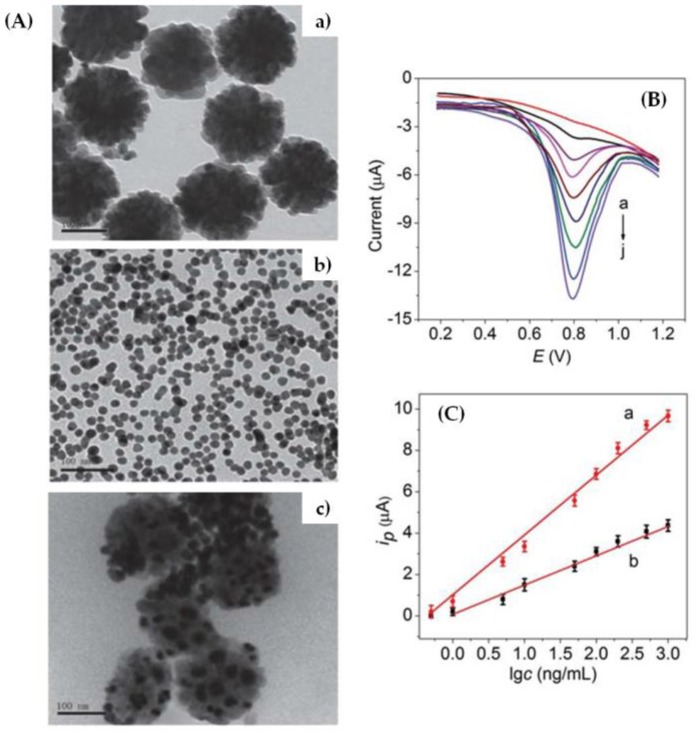
(**A**) TEM images of (**a**) Fe_3_O_4_, (**b**) gold nanoparticles, and (**c**) Fe_3_O_4_@gold nanocomposites; (**B**) square wave voltammetry measurements of methyl parathion with different concentrations between (**a**) 0.5 and (**j**) 1000 ng mL^−1^; and (**C**) calibration curves obtained with (**a**) and without (**b**) gold nanoparticles (Reproduced from Zhao et al. [8] with permission of The Royal Society of Chemistry).

Another organophosphorous pesticide, the methyl parathion, was determined by a sensitive and selective enzymatic biosensor using hydrolase and a uniform nanocomposite based on magnetic Fe_3_O_4_ (average diameter of 120 nm) and gold nanoparticles (diameter ~13 nm) [8]. The cysteamine was also used as a linker and transmission electron microscopy (TEM) images of such nanocomposites could be visualized in Figure 1A. The biosensor could be reusable for continuous measurement and the advantage of using hydrolase is its no poisoning by the organophosphorous pesticide [8].

**Figure 2:**
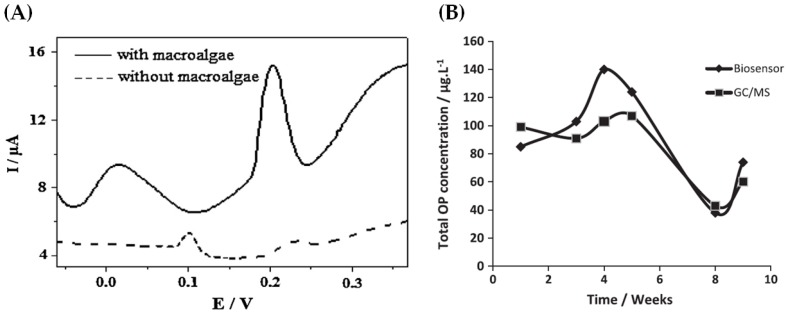
(**A**) current results of biosensor with and without macroalgae; and (**B**) total concentration of methyl parathion obtained using the biosensor and SPME-GC/MS over 10 weeks in the same collecting point on a Brazilian lake (Reprinted from Nunes et al. [14], Copyright (2014), with permission from Elsevier).

The biosensor shows a rapid response and high selectivity for the detection of methyl parathion, with a linear range from 0.5 to 1000 ng mL^−1^ and a limit of detection of 0.1 ng mL^−1^. As shown in Figure 1B, the current increased with the increase of pesticide concentration, with proportionality between them (Figure 1C). With gold nanoparticles, the biosensor displays a higher sensitivity and wide linear range, as showed in Figure 1C, which could be attributed to the high conductivity, high catalytic efficiency, and excellent biocompatibility of gold nanoparticles. Other organophosphorous pesticides such as dimethoate, monocrotophos, and malathion were also detected by the hydrolase biosensor and no interferences with the detection of methyl parathion were observed due to the high selectivity of the enzyme to P-S containing pesticides [8]. Another enzymatic biosensor (acetylcholinesterase) based on amperometry and using a graphite working electrode and macroalgae (*Cladophoropsis membranacea*) as a functional immobilization support was proposed to the detection of methyl parathion in natural water samples from a contaminated lake, obtaining limits of detection between 1.5 and 1.8 ng mL^−1^ [14]. The use of hydrolyzed macroalgae as immobilization matrix reduced both enzyme amount and substrate concentration, thus reducing the biosensor cost [14]. On the other hand, the incorporation of the macroalgae as the immobilization support resulted in a synergic effect, leading to enhanced enzyme stability and sensitivity of the biosensor. Figure 2A shows the results of a comparative electrochemical study to demonstrate the beneficial characteristic of macroalgae powder on the sensor surface; the presence of macroalgae powder shifted the working potential to lower values and also increases the current intensity about three times. In order to compare the biosensor potential to detect methyl parathion on natural water samples, solid-phase microextraction-gas chromatography/mass spectrometry (SPME-GC/MS) was also used as the reference analytical technique, whose limit of detection was found as 14.5 ng mL^−1^. From Figure 2B, it is possible to observe a good correlation between SPME-GC/MS and the biosensors, when the concentration of methyl parathion was obtained over 10 weeks (statistical deviation lower than 20%); the concentration varied over time since the pollution was not constant [7].

**Figure 3:**
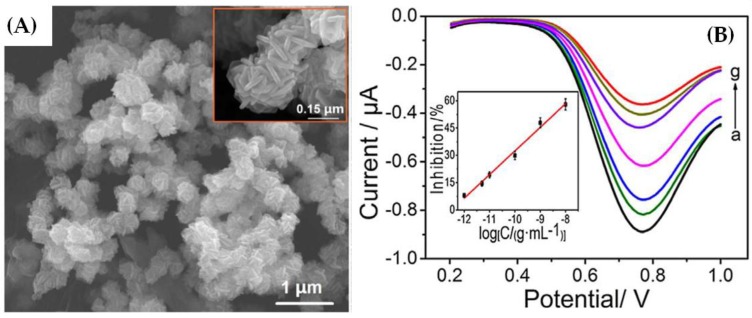
(**A**) SEM images of NiCo_2_S_4_; and (**B**) differential pulse voltammetry response of the biosensor for concentrations of methyl parathion between 0 and 10 ng mL^−1^ (Reprinted from Peng et al. [15], Copyright (2017), with permission from Elsevier).

Recently, an improved limit of detection for methyl parathion (0.42 pg mL^−1^) with a wide linear range (1.0 pg mL^−1^ to 10 ng mL^−1^) was obtained through an electrochemical biosensor based on carbon paste electrode and reticulated hollow spheres structures of nickel cobaltite (NiCo_2_S_4_) material [15]. The lower limit of detection could be due to the improved electron transfer rate provided by the NiCo_2_S_4_ material since it can offer rich reaction active sites and high electronic conductivity [15]. Scanning electron microscopy (SEM) image of NiCo_2_S_4_ material is shown in Figure 3A, where many ball-like spheres are observed. In the inset of Figure 3A, it is possible to observe the reticulated and assembled rod-like structures of relatively uniform lengths (~150 nm). In Figure 3B, the differential pulse voltammetry response of the biosensor was observed for concentrations of methyl parathion between 0 (curve a) and 10 (curve g) ng mL^−1^. In addition, the biosensor retained 92% of its initial peak current response after two weeks at 4 °C, which indicated a long-term stability.

In another work, the detection of methyl parathion by an electrochemical biosensor provided a highly improved limit of detection at femtogram level (5 fg mL^−1^) [16]. The biosensor was constructed using a nanoporous carbon paste electrode with chitosan, gold nanoparticles, and Nafion, which was used as a protective membrane and employing acetylcholinesterase as the recognition element. A high affinity to the substrate of acetylcholinesterase was observed producing a fast biosensor response due to the excellent electron transfer rate and biocompatibility of gold nanoparticles and chitosan [16]. A whole-cell optical biosensor based on *Sphingomonas* sp. cells was recently proposed as an interesting alternative for methyl parathion detection [17]. The optical biosensor used silica nanoparticles functionalized with polyethyleneimine, which provided an enhanced storage stability (180 days with a retention of 85% of activity when stored at 4 °C), mainly due to the protective covering provided around the cells also reducing the leaching of the hydrolytic enzyme. This excellent storage stability enables many routine applications such as decentralized field testing, screening, and rapid detection of multiple samples, avoiding the time-consuming preparation of a fresh biosensor [17]. In addition, the optical biosensor is reusable, since, after ten repeated reactions, 81% of the enzyme activity was maintained.

For chlorpyrifos detection in river water samples, nanoparticles based on iridium oxide were used in disposable enzymatic biosensor with tyrosinase based on low-cost screen printed carbon electrodes [18]. A linear biosensor response (0.01–0.1 µM) and low limit of detection (3 nM) were obtained, which could be attributed to the high conductivity of iridium oxide nanoparticles and efficiency of tyrosinase. To demonstrate the applicability of the biosensor, recovery tests were performed in river water samples with the addition of 0.1 µM of chlorpyrifos and recoveries of 90 ± 9.6% were observed with a residual standard deviation (RSD) lower than 10% (*n* = 3). Improved limit of detection for chlorpyrifos (0.13 pM) and wide linear range (0.01 nM–0.1 µM) were obtained by an enzymatic (acetylcholinesterase) biosensor based on a boron-doped diamond electrode with gold nanoparticles and carbon spheres (average diameter of 500 nm) [19]. This improved limit of detection could be due to the larger surface of nanocomposite (gold nanoparticles-carbon spheres), which improves the adsorption of acetylcholinesterase, thus enhancing its activity and facilitating electrocatalysis [19]. In addition, the improved characteristics of carbon spheres such as tunability of particle size and shape, homogeneity of particle size, and porous nanostructure for large loading of guest molecules are advantageous for the immobilization of enzymes [19]. Recently, an electrochemical aptasensor based on a novel composite film constituted by carbon black and graphene oxide@Fe_3_O_4_ was proposed for the detection of chlorpyrifos in real vegetable samples, obtaining a limit of detection of 94 pM [20]. This low limit of detection could be attributed to the amplification of current signal of the aptasensor due to the high specific surface area of functionalized carbon black, its ideal dispersability, and good electrical conductivity as well as facility to capture more graphene oxide@Fe_3_O_4_ [20]. The analytical performance of the aptasensor for chlorpyrifos detection was compared with enzymatic biosensors, and it was observed that the aptasensor displayed a broader linear range (0.29 nM–0.29 mM) and lower limit of detection (94 pM) than enzymatic (acetylcholinesterase) biosensors (linear range from 0.05 to 10 mM and limit of detection of 0.4 nM [74] and linear range from 5 nM to 1 µM and limit of detection of 3 nM [75]). The selectivity of the aptasensor was also tested by determining interfering substances such as carbofuran, methyl parathion, carbaryl, and acetamiprid and the current signal variation was less than 5% of that without interferences, suggesting a good selectivity.

For the detection of dichlorvos in real samples such as apples, a fast and simple fluorescence biosensor was proposed using a bi-enzyme (acetylcholinesterase and choline oxidase) system, quantum dots, as well as acetylcholine as substrate [22]. A linear range of 4.49–6780 nM and a low limit of detection (4.49 nM) was obtained, which is lower than the maximum residue level for dichlorvos (0.45 µM) in imported fruits and vegetables established by the European legislation [76]. In addition, good reproducibility was observed (RSD = 2.2%) after the evaluation of biosensor response in six different assays. Another enzymatic biosensor based on acetylcholinesterase-zinc oxide modified platinum electrode was reported for the detection of dichlorvos in orange samples with a limit of detection of 12 pM [23]. This low limit of detection was attributed to the high electron transfer rate and good biocompatibility properties of zinc oxide nanospheres. In addition, the acetylcholinesterase enzyme was successfully reactivated using PAM (4.0 mM solution) for 30 min, recovering the catalytic activity of enzyme; the reactivation efficiency was found to be 96.3% of original catalytic activity, which confirms the possibility to repeatedly use the biosensor for dichlorvos determination. Also for dichlorvos detection, an improved limit of detection (0.3 pM) was obtained with an acetylcholinesterase biosensor based on ionic liquids-gold nanoparticles-porous carbon composite matrix in a linear range between 0.45 pM and 4.5 nM [15]. A good biosensor reproducibility (RSD = 6.5%) was also obtained with good stability since, after 21 days, the current density was inclined to be constant and returned almost 92% of response [24]. The combination of porous carbon, gold nanoparticles, and ionic liquids as an immobilization matrix for acetylcholinesterase produces a synergic effect, which improves the biosensor performance including the improvement of enzyme adsorption, retaining enzyme activity, and enhancing the sensitivity of analytical response [24]. The inhibited acetylcholinesterase was reactivated by PAM (5.0 mM solution) for 15 min, and it was found that the enzymatic biosensor could regenerate 91.7% of its original activity.

#### 2.1.2. Other Pesticides

Acetamiprid has been detected in real environmental samples such as fresh surface soil samples by colorimetric aptasensors [25] and water samples by impedimetric aptasensors [26]. With the colorimetric aptasensor, a linear range between 75 nM to 7.5 µM and a limit of detection of 5 nM were found while a larger linear range (50 fM to 10 µM) and lower limit of detection (17 fM) were obtained with the impedimetric aptasensor. In that biosensor, gold nanoparticles, multi-walled carbon nanotubes (MWCNT), and reduced graphene oxide nanoribbons were used as composite for supporting the acetamiprid aptamer at the electrode surface, which could be responsible for higher electron transfer and better biosensor analytical performance [26]. Similar limit of detection (33 fM) was obtained by an aptasensor based on silver nanoparticles anchored on nitrogen-doped graphene oxide nanocomposite constructed for the detection of acetamiprid in wastewater samples [27]. The low limit of detection could be due to excellent electrical properties and large surface area of silver nanoparticles and nitrogen doped graphene, which display more effective electron transfer and high loading capacity than the nanomaterials alone. In addition, the nanocomposite constitutes an ideal support for the immobilization of aptamer, which promotes the amplification of response signal and further detection of acetamiprid with high sensitivity [27]. The selectivity of the aptasensor was also investigated by comparison of results of carbaryl, chlorpyrifos, imidacloprid, methyl parathion, and pentachlorophenol, and the response signals to the other pesticides were negligible, indicating the high selectivity of the aptasensor to acetamiprid.

Another pesticide (atrazine) was analysed in environmental samples such as crop samples [29] and seawater/riverine water samples [30]. A label-free electrochemical (voltammetric) immunosensor based on gold nanoparticles and a disposable and label-free electrochemical immunosensor based on field effect transistor with single-walled carbon nanotubes (SWCNT) were respectively used. The FET immunosensor displays a lower limit of detection of 0.001 ng mL^−1^ vs. 0.016 ng mL^−1^ for the voltammetric immunosensor, and both lower to the legal limits (0.1 ng mL^−1^). Recently, a highly sensitive impedimetric aptasensor based on interdigitated electrodes and microwires formed by platinum nanoparticles was proposed to the selective detection of acetamiprid and atrazine in real water samples [28]. Improved limits of detection of approximately 0.22 pg mL^−1^ (1 pM) and 2.2 pg mL^−1^ (10 pM) were observed for acetamiprid and atrazine, respectively, which could be associated with the enhanced charge transfer occurring on the biosensing platform. Another atrazine biosensor (electrochemical immunosensor) was also suggested using a new recognition element constituted by a recombinant M13 phage/antibody complex and magnetic beads functionalized with protein G [31]. The biosensor provided an enhanced limit of detection (0.2 pg mL^−1^), which could be attributed to the high sensitivity of phage/antibody complex and reaction kinetics of the covalent assembly of magnetic beads with protein G [31].

Enzymatic biosensors were also used for the detection of pirimicarb, which is a carbamate insecticide, using enzymatic (acetylcholinesterase and laccase) biosensors [32,33]. The enzymatic biosensor based on laccase enzyme used MWCNT on composite carbon paste electrode for the detection of pirimicarb with a limit of detection of 43 µg mL^−1^ [32]. In addition, the stability was tested by storing the biosensor at 4 °C during one month and 92.4% of its initial current response was retained, demonstrating the preservation of the enzyme integrity during an acceptable period of time [32]. The acetylcholinesterase biosensor used Prussian blue-MWCNT screen-printed electrodes and an improved limit of detection (53.2 ng L^−1^), high sensitivity (21.97 µA mM^−1^ cm^−2^), and good stability with 15% activity loss of its initial current response after 20-day storage period were obtained, indicating that the Prussian blue-MWCNT provided favourable conditions for the enzyme to maintain its biological activity to a large extent [33]. Another carbamate insecticide (carbofuran) was detected by an electrochemical (square wave voltammetric) biosensor based on acetylcholinesterase immobilized onto iron oxide-chitosan nanocomposite [34]. A limit of detection of 3.6 nM and linear detection range between 5 and 90 nM were obtained. A good reproducibility was also reported with RSD values of 5.4% after applying five freshly prepared enzymatic biosensors to the detection of a 10 nM carbofuran solution. Another electrochemical enzymatic biosensor for carbofuran was prepared with a 3D graphene oxide network and MWCNT composite, which improves the electron the electron transfer rate between carbofuran and electrode surface [35]. After storing 20 days at 4 °C, the biosensor retains 87% of its initial response, showing a good stability, and, when tested in real water samples fortified with different amounts of carbofuran, recoveries of 102.38 ± 2.05% were obtained, demonstrating the applicability of the biosensor [35]. A better limit of detection was obtained from an electrochemical biosensor fabricated through immobilization of acetylcholinesterase on NiO nanoparticles-carboxylic graphene-Nafion modified glassy carbon electrode for the detection of carbofuran (0.5 pM) together with pesticides methyl parathion and chlorpyrifos (limit of detection of 0.05 pM) [21]. It was found that the NiO nanoparticles conjugated with carboxylic graphene improve the electron transfer and decrease the oxidation peak potential, which is advantageous for avoiding interference from other electroactive species in a biological matrix [21]. When applied to real samples such as lake water samples, the recoveries observed by the biosensor were in the range of 93.0–105.2%. The carbamate pesticide carbaryl was also detected by electrochemical enzymatic (acetylcholinesterase) biosensors based on gold electrodes with cysteamine self-assembled monolayer [36], on interdigitated array microelectrodes with chitosan [37], and glassy carbon electrodes with MWCNT and graphene oxide nanoribbons [38], where the better limit of detection (1.7 nM) was obtained. Such improved analytical performance could be due to the covalent immobilization of acetylcholinesterase on the modified electrode with MWCNT and graphene oxide nanoribbons, which provide enhanced enzymatic activity. Irgarol 1051 is an herbicide commonly found in seawater due to its use in antifouling paints and an amperometric immunosensor based on gold screen-printed electrodes was recently developed for its detection and continuous monitoring [77]. The biosensing system provided a limit of detection of 0.15 ± 0.09 nM directly measured in seawater, and it was able to generate successive measurements (5 cycles) using the same chip through an easy regeneration with NaOH solution, obtaining a good repeatability (coefficient of variation, CV of 7%) [77].

### 2.2. Pathogens

The presence of pathogens in environmental matrices, and mainly in water compartments, could constitute a serious danger for human health and some biosensors were recently proposed for their environmental monitoring. For example, rapid and specific optical biosensors based on surface plasmon resonance were proposed for the detection of metabolically active *Legionella pneumophila* in complex environmental water samples [40,41]. In one work, the principle of detection was based on the recognition of bacterial RNA by the RNA detector probe immobilized on the biochip gold surface [40]. Streptavidin-conjugated quantum dots were used for signal amplification and the detection time was about three hours, suggesting the viability of the biosensing system for effective detection of bacteria in a range of 10^4^–10^8^ CFU mL^−1^ [40]. In the second work, the gold substrate was functionalized with a self-assembled monolayer of protein A and an antibody solution against *L. pneumophila* [41]. A limit of detection of 10^3^ CFU mL^−1^ was obtained and the biosensor was able to detect *L. pneumophila* in contaminated water samples in 30 min since any labelling was needed and the immobilization of antibodies in biosensor surface by protein A in a highly oriented manner was stable and effective [41]. A disposable electrochemical immunosensor based on screen-printed carbon electrodes with magnetic (Fe_3_O_4_) nanoparticles and polydopamine was proposed for *L. pneumophila* detection, where bacteria were sandwiched using the antibody labelled with horseradish peroxidase [42]. A good limit of detection (10^4^ CFU mL^−1^), good selectivity, and stability were obtained. The selectivity was tested against other bacteria (*Escherichia coli*, *Enterococcus faecalis*, *Pseudomonas aeruginosa*, *Aeromonas*, and *Salmonella*) and no interference was observed. The storage stability was evaluated after storing the biosensor at 4 °C for 30 days and no significant differences in the measured amperometric signals were apparent. An improved limit of detection (10 CFU mL^−1^) was recently reached with an electrochemical biosensor based on grating coupling screen plasmon resonance principle for *L. pneumophila* detection [43]. The good specificity of the biosensor was reported using *E. coli* with only 1% of the SPR response obtained. In another work, *E. coli* was detected in ground water sources by a whole cell imprinting biosensor based on optical (surface plasmon resonance) and piezoelectric (quartz crystal microbalance) principles, providing real-time detection capabilities and total detection times of 1 h or less [44]. A polymerizable form of histidine (*N*-methacryloyl-l-histidine methyl ester) was used as a recognition element and immobilized on gold surfaces, obtaining similar recognition to natural antibodies. Limits of detection of 3.72 × 10^5^ and 1.54 × 10^6^ CFU mL^−1^ were respectively obtained for the optical and piezoelectric biosensors. Improved limit of detection (70 CFU mL^−1^) was obtained by a whole cell based microcontact imprinted capacitive biosensor based on gold electrodes for the detection of *E. coli* in real river water samples [45]. The low limit of detection could be due to the sensitivity of capacitive system and chemical/physical stability provided by the polymeric material (polymerizable form of histidine, also used in this work). In addition, the selectivity of the capacitive biosensor to *Bacillus subtilis*, *Staphylococcus aureus*, and *Salmonella paratyphi* was evaluated and the responses were approximately 70% lower than that of *E. coli*. In a recent work, an electrochemical immunosensor was reported for *E. coli* detection using a polydopamine surface imprinted polymer sensing surface with nitrogen-doped graphene oxide quantum dots [46]. An excellent limit of detection (8 CFU mL^−1^) was obtained, which could be attributed to the enhanced photo-electrochemical electron transfer, photoluminescence of graphene quantum dots, and specific binding between the bacteria and polyclonal antibodies [46]. Also in airborne dust, specifically during Asian dust events, the detection of pathogenic bacteria (*B. subtilis*) was reported through an electrochemical immunosensor based on SWCNT-gold electrodes [47]. A limit of detection of 10^2^ CFU mL^−1^ was obtained in a detection range between 10^2^ and 10^10^ CFU mL^−1^ while the response time was 10 min. In addition, the specificity of the biosensor was confirmed using other microorganisms (*S. aureus*, *Flavobacterium psychrolimnae*, and *Aquabacterium commune*) as a control between 0 and 10^6^ CFU mL^−1^, and it was found that the electrical resistances obtained were similar than that obtained by the immobilized antibody.

### 2.3. Potentially Toxic Elements

The pollution of natural water environment by heavy metals and respective ions can cause severe hazards to human health and portable, low cost, and fast heavy metal analyses are a priority issue worldwide. Mercury ions (Hg^2+^) were used as model target for testing a DNA optical biosensor for the detection of heavy metal ions, which are highly toxic and ubiquitous pollutants in the environment [48]. The biosensor was portable, low-cost, and fast with in situ screening of Hg^2+^ in less than 10 min in natural waters. The principle of detection is based on the ability of some metal ions in selectively bind to some bases to form stable metal-mediated DNA duplexes; in the case of Hg^2+^, they are capable of selectively coordinating thymine bases to form stable thymine-Hg^2+^-thymine complexes [48]. A limit of detection of 1.2 nM was obtained in a detection range between 0 and 1000 nM, which is lower than the maximum value demanded by the United States Environmental Protection Agency (10 nM) [78]. The selectivity of the biosensor was tested for other metal cations such as Zn^2+^, Cu^2+^, Ni^2+^, Pb^2+^, and Cr^2+^ at concentrations up to 20 µM and no significant response (lower than 15% in comparison to the response to Hg^2+^) was observed. Recently, a surface enhancement Raman spectrum (SERS) biosensor was proposed for simple and sensitive detection of Hg^2+^ between 1 pM and 100 nM using magnetic substrate (CoFe_3_O_4_@Ag) conjugated with single-stranded DNA and SWCNT [51]. The SWCNT serve as Raman labels to produce characteristic Raman peaks, which are the signal to quantitatively detect Hg^2+^ [28]. An improved limit of detection (0.84 pM) was found, which could be due to the magnetic aggregation of the biosensor by the magnetic substrate, enhancing the Raman intensity and thus the biosensor sensitivity. A femtomolar limit of detection (3 fM) was provided by an electrochemical biosensor for Hg^2+^ based on tunable vertically aligned SWCNT, which displayed superior properties such as large specific area, high electrical conductivity, and excellent substrate binding strength [50]. In addition, due to the reversibility of π–π interactions provided by SWCNT, the biosensor interface was regenerated for 50 consecutive measurements without significant signal loss.

**Figure 4:**
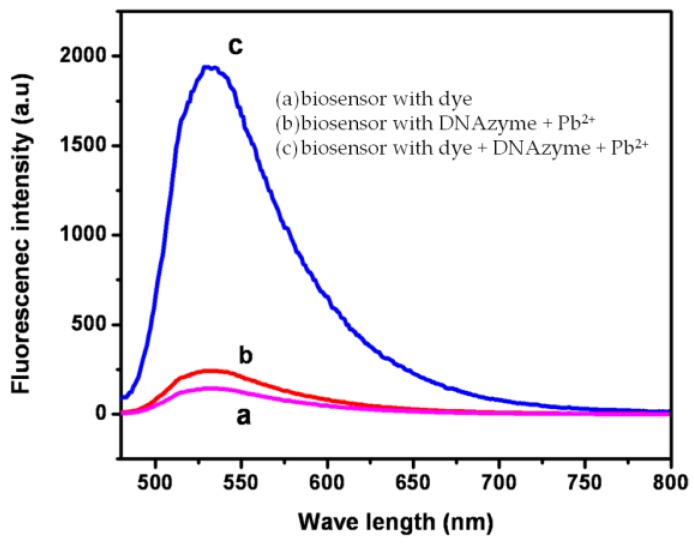
Fluorescence emission spectra obtained with the biosensor with the dye (curve a), with the DNAzyme and Pb^2+^ (curve b), and with the dye, DNAzyme/carboxylated magnetic beads, and Pb^2+^ (curve c); λ_ex_ = 490 nm and λ_em_ = 530 nm (Reprinted from [52] with kind permission from Springer).

Recently, two optical biosensors based on fluorescence were proposed for the detection of Pb^2+^ in water samples (pond and lake water samples) using DNAzymes/carboxylated magnetic beads [52] and DNA aptamers [54]. Limits of detection of 5 nM and 61 nM were, respectively, obtained by the DNAzymes-based and DNA aptamers-based biosensors, with respective detection linear range from 0 to 50 nM and from 100 to 1000 nM. The better limit of detection provided by the DNAzymes-based biosensor could be due to the use of label free specific dye (SYBER Green I), which was intercalated with double stranded DNA, displaying strong fluorescence intensities, as shown in Figure 4. In this figure, the absence of fluorescence intensity of the biosensor only with the dye (curve a) and with the DNAzyme + Pb^2+^ (curve b) is observed, but, upon addition of the dye + DNAzyme + Pb^2+^, the fluorescence intensity increases, demonstrating the biosensor sensitivity toward Pb^2+^.

A multi-analyte biosensor was proposed for the determination of Pb^2+^ and Cd^2+^ using mesoporous carbon nitride/self doped polyaniline nanofibres [79]. Limits of detection of 0.2 nM and 0.7 nM were attained for Pb^2+^ and Cd^2+^. Similar limits of detection of 0.33 nM and 0.24 nM were obtained for Pb^2+^ and Cd^2+^, respectively, using a wireless biosensor based on magnetoelastic principle, which allows their real-time monitoring in remote locations [80]. The biosensor was functionalized with bovine serum albumin on gold electrodes, and the precipitation induced contributes to an increase of mass loading and decrease of resonance frequency, which is proportional to the concentration of heavy metal ions. Good limits of detection of 0.33 nM and 0.24 nM were obtained for Pb^2+^ and Cd^2+^, respectively.

For the detection of Zn^2+^, a recent electrochemical biosensor was proposed using paper-based microfluidic channels with reduced graphene oxide and chitosan [81]. The biosensor is considered as a lab-on-chip system due to the occurrence of immobilization, separation, rinse, and high-throughput analysis on the sensing platform. A large linear range (0.1–7000 nM) and low limit of detection (0.03 nM) were obtained. The biosensor was able to detect Zn^2+^ in complex environmental samples since it was found as selective when the other seven cations (Cu^2+^, Fe^3+^, Cd^2+^, Hg^2+^, Mn^2+^, Mg^2+^, and Ag^2+^) were examined; the biosensor exhibited a 3-fold higher current signal for Zn^2+^, as compared to other metal ions with 100-fold higher concentration than Zn^2+^ [81].

### 2.4. Toxins

Harmful toxins such as brevetoxins and microcystins are produced from the algal blooms of cyanobacteria provided by the eutrophication of aquatic systems, and reliable and cost-effective systems are thus needed for the early detection of such toxins. An electrochemical aptasensor was applied to the sensitive detection of brevetoxin-2, a marine neurotoxin, using gold electrodes functionalized with cysteamine self-assembled monolayer [55]. A limit of detection of 106 pg mL^−1^ was obtained and good selectivity to brevetoxin-2 was observed against other toxins from different groups such as okadaic acid and microcystins [55]. The feasibility of the aptasensor to detect brevetoxin-2 in real samples was performed by analysing shellfish and good recoveries were obtained (102–110%), indicating no interferences from the shellfish matrix on the aptasensor response. In another work, a portable cardiomyocyte-based biosensor was proposed for saxitoxin and brevetoxin-2 detection obtaining limits of detection of 0.35 ng mL^−1^ and 1.55 ng mL^−1^, respectively [56]. Cardiomyocytes were grown on microelectrode arrays, which provide rapid and real-time monitoring of pathogens (5 min). The principle of detection is based on the changes of electrophysiological function induced by the ion channel toxins. Microcystin-LR was detected in water matrices by an electrochemical impedance spectroscopy immunosensor based on graphene with a limit of detection of 50 pg mL^−1^, good reproducibility (CV of 6.9%) and repeatability (CV of 3.6%) results [58]. In addition, the stability was tested by storing the biosensor at 4 °C from one to two weeks and the responses were reduced to 91.6 and 81.3% of initial levels, respectively, which could be attributed to the ability of surface-attached biomolecules to provide slow disintegration during the storage [58]. Improved limit of detection (5 pg mL^−1^) in a linear range between 0.01 and 20 ng mL^−1^ was obtained with an electrochemical immunosensor based on gold electrodes modified with a composite based on molybdenum disulfide/gold nanorods for the detection of microcystin-LR in real lake water samples [59]. The low limit of detection could be due to the synergistic effect between molybdenum disulfide and gold nanorods, which promoted a larger surface area enhancing the electrochemical performance and mainly the electrical conductivity. In addition, the biosensor has the advantage to be regenerated with glycine-HCl solution (pH 3.0) in order to dissociate the antigen–antibody complex; 90.56% of the initial response value could be restored after five assay runs, showing good reusability [59].

Biosensors were also reported for the detection of toxin okadaic acid in real algal, seawater, and shellfish samples [61,62,63]. For okadaic analysis in algal and seawater samples, a multiplex surface plasmon resonance biosensor was proposed and also for the detection of saxitoxin and domoic acid [61]. For the detection of okadaic acid in algal cells, a simple sample preparation procedure was employed where the cell lysis and releasing of the toxins were performed with glass beads followed by centrifugation and filtering the extract. Limits of detection of 0.82, 0.36, and 1.66 ng mL^−1^ were obtained for saxitoxin, okadaic acid, and domoic acid, respectively [61]. The biosensor was stable over a period of eight weeks with a little variation in response after the daily regeneration of biosensor with fresh antibody solutions. A total of 256 seawater samples were collected from European countries and 47%, 59%, and 61% of them tested positive by the biosensor for saxitoxin, okadaic acid, and domoic acid, respectively, with toxic samples found in Spain and Ireland [61]. The authors referred that the developed multiplex immunological methods could be used as early warning monitoring tools for a variety of other marine biotoxins in seawater samples.

**Figure 5:**
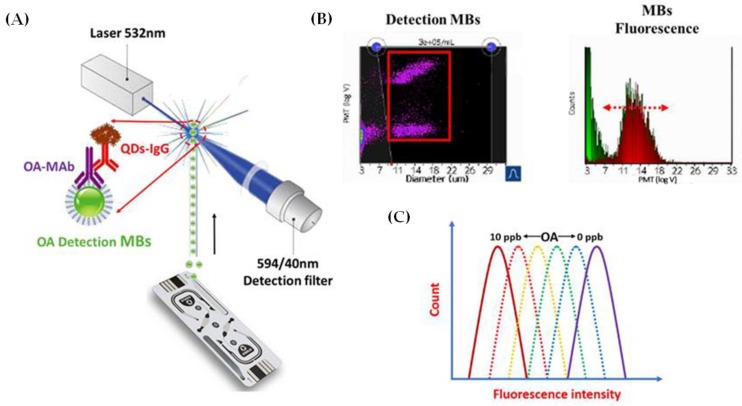
(**A**) working principle of fluorescence immunosensor for the detection of okadaic acid; (**B**) draw gate for the detection of magnetic beads and measure the fluorescence intensity; and (**C**) typical fluorescence intensity curves of fluorescent immunosensor under different okadaic acid concentrations. OA: okadaic acid; MBs: magnetic beads; MAb: monoclonal antibodies; QDs-IgG: quantum dots labelled with secondary antibodies IgG (© 2017, Pan et al. [63]. Originally published in “A novel quantum dot fluorescence immunosensor based on magnetic beads and portable flow cytometry for detection of okadaic acid” under Creative Commons 4.0 license).

Disposable electrochemical immunosensors based on field effect transistors with graphene were also proposed for okadaic acid detection in real seawater samples [62]. Improved limit of detection (0.05 ng mL^−1^), wide working range (0.05–300 ng mL^−1^), and good reproducibility (0.54–2.19%) were obtained, which could be attributed to the fast electron mobility, high current density, and large surface area provided by graphene [62]. Similar limit of detection (0.05 ng mL^−1^) was obtained with a high sensitive fluorescence immunosensor based on carboxylic acid modified magnetic beads and CdTe quantum dots for the detection of okadaic acid in mussel samples [63]. Figure 5 shows the working principle of fluorescence immunosensor (Figure 5A,B) and the typical fluorescence curves of the immunosensor (Figure 5C). The detection of okadaic acid in real samples was completed within one hour, attributed to the use of magnetic beads, improving the sensitivity of the immunosensor and eliminating the matrix effect from the mussel extract.

Another toxin, the domoic acid, was also detected in seawater samples with disposable carbon nanotube field effect transistor immunosensors [64]. Good reproducibility (0.52–1.43%) and low limit of detection (10 ng mL^−1^) were found in a working range between 10 and 500 ng mL^−1^. An improved limit of detection (0.1 ng mL^−1^) was obtained for domoic acid by an underwater biosensor based on surface plasmon resonance principle [65]. The advantage of such biosensor was the in situ quantification of domoic acid in seawater due to its incorporation in an oceanographic vessel. 

### 2.5. Endocrine Disrupting Chemicals

As an endocrine disrupting chemical, bisphenol A was detected in water samples by aptasensors based on fluorescence principle with functionalized aptamers (by fluorescein amidite) and gold nanoparticles [66] and based on evanescent-wave optical fibre [67]. The evanescent-wave optical fibre aptasensor was portable and found as fast, cost-effective, sensitive, and selective for bisphenol A detection in water samples with the advantage of no requirement of any pre-concentration and treatment steps [67]. In addition, the aptasensor can be reused by regeneration with 0.5% sodium dodecyl sulphate (SDS) solution for 90 s and further washing with a phosphate buffered saline (PBS) solution (pH 7.2) for over a hundred assay cycles without any significant loss of the aptasensor performance [67]. Similar limits of detection were found (0.1 and 0.45 ng mL^−1^) in both optical biosensors, where the probe DNA molecule, which is the complementary sequence of a small part of the bisphenol A aptamer, was adsorbed in gold nanoparticles surface by electrostatic interaction [66] and was immobilized on fibre surface covalently [67]. Recently, another aptasensor based on fluorescence was proposed for the detection of bisphenol A in river water samples using molybdenum carbide nanotubes [68]. A low limit of detection of 0.23 ng mL^−1^ was obtained with such a label-free, inexpensive, and easy to use aptasensor. The specificity of the aptasensor was evaluated by analysing other molecules with similar structures than bisphenol A (e.g., 4,4′-biphenol, bisphenol AF, and 4,4′-sulfonyldiphenol) and only background signals were found for these molecules, displaying the high specificity for bisphenol A.

Another endocrine disrupting chemical, the 4-nonylphenol, was recently analysed in seawater samples by a disposable and label-free electrochemical immunosensor based on a field effect transistor with SWCNT [69]. The immunosensor displays a high reproducibility (0.56 ± 0.08%), average recovery between 97.8% and 104.6%, and low limit of detection (5 µg L^−1^), which is lower than the recommended maximum concentration defined by the United States Environmental Protection Agency (EPA) as 7 µg L^−1^ [82]. The biosensor could be used to detect hazardous priority substances in seawater samples such as 4-nonylphenol, even at low concentrations and through a simple and low-cost methodology.

**Figure 6:**
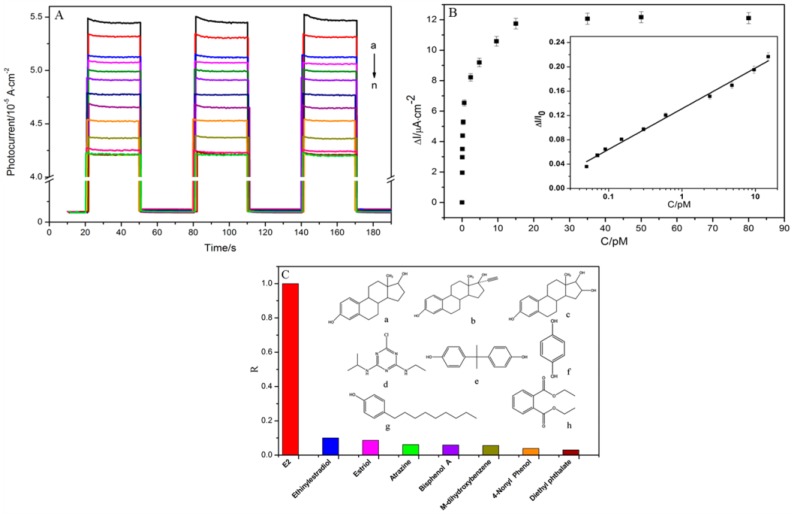
(**A**) photocurrent change of the aptasensor in different concentrations of 17β-estradiol from 0 (response a) to 80 pM (response n); (**B**) curve of ΔI corresponding to the concentration of 17β-estradiol from 0 to 80 pM (ΔI was calculated by I_0_ subtracting I, where I_0_ and I are the photocurrent before and after incubation of 17β-estradiol). The inset is the linear relationship between ΔI/I_0_ and the logarithm of 17β-estradiol concentrations from 0.05 to 15 pM; and (**C**) selectivity of the assay for 17β-estradiol on the aptasensor. The inset are the structural formulas of 17β-estradiol and the tested interferents (Reprinted with permission from Fan et al. [70]. Copyright (2014) American Chemical Society).

17β-estradiol was detected in environmental water samples (lake water) by aptasensors [70,83]. With a photo-electrochemical aptasensor based on CdSe nanoparticles modified with ordered vertically aligned TiO_2_ nanotube arrays, a femtomolar level (limit of detection of 33 fM) and highly selective detection of 17β-estradiol was performed using an anti-17β-estradiol aptamer as biorecognition element [70]. The specific recognition reaction between the 17β-estradiol and aptamer leads to the in situ formation of complexes on the surface of the photo-electrochemical sensing interface, which increases the steric hindrances for the diffusion of the electron donors, thus leading to the decrease of the photocurrent. Figure 6A shows the variation of the photocurrent in various concentrations of 17β-estradiol, observing its gradual decrease with higher concentration until 15 pM. A wide linear range (0.05–15 pM) was observed in the inset of Figure 6B with a correlation coefficient of 0.992. The outstanding selectivity of the biosensor to 17β-estradiol was reported after testing the biosensor to other seven endocrine disrupting compounds that have similar structure coexisting with 17β-estradiol including estriol, ethinylestradiol, atrazine, m-dihydroxybenzene, bisphenol A, 4-nonylphenol, and diethyl phthalate, as observed in Figure 6C [70]. The high selectivity to 17β-estradiol could be attributed to the tubular microstructure of sensing interface, excellent photoelectrical activity, large packing density of aptamer, and high affinity of the aptamer to 17β-estradiol [70].

### 2.6. Other Environmental Compounds

Due to the high frequency of harmful algal blooms, new, rapid, and reliable analytical methodologies have been required to their early detection and monitoring. Biosensors have been developed for the detection of algal RNA due to the excellent sensitivity and specificity of nucleic acid probes to their complementary binding partners [84,85]. An electrochemical genosensor based on screen printed gold electrode was recently reported for the enhanced selective and sensitive detection of RNA from 13 harmful algal species; the genosensor could discriminate RNA targets from environmental samples (spiked seawater samples) containing 10^5^ cells, considered as the limit of detection [84].

Halogenated compounds were also detected by environmental biosensors, for example, by a fluorescence-based enzymatic biosensor proposed for the monitoring of 1,2-dichloroethane, 1,2,3-trichloropropane, and γ-hexachlorocyclohexane in water samples with pH ranging from 4 to 10 and temperature from 5 to 60 °C [86]. Limits of detection of 2.7, 1.4, and 12.1 mg L^−1^ were obtained for 1,2-dichloroethane, 1,2,3-trichloropropane, and γ-hexachlorocyclohexane, respectively. When tested in real conditions, the biosensor was used for rapid quantification of 1,2-dichloroethane contamination in water with the ability for mapping the contamination distribution through GPS [86].

## 3. Future Perspectives and Concluding Remarks

Although environmental biosensors can be constructed using the improved characteristics of nanomaterials and novel nanocomposites, an increased attention has been focused on the in situ and real-time monitoring of pollutants by other technologies. The drones, also known as unmanned aircraft systems (UAS), unmanned aerial vehicles (UAV), and remotely piloted aircraft (RPA), refer to aircraft, which fly without a human operator onboard and they could be found in the areas of surveillance, reconnaissance, and military missions using sensing instruments such as electro-optical sensors, infrared sensors, and synthetic aperture radars. Recently, environmental monitoring has been a field of interest for the application of drones mainly in the monitoring of water and air quality, the surveillance of agriculture, and volcano gas measurements, as reported in some daily news, and a few scientific articles are present in the current literature. For example, a portable and sensitive whole cell biosensor was recently incorporated in a UAV (Phantom 2, Shenzhen, China) for air quality control and water pollution monitoring in remote locations [87]. The maximum flight time is approximately 20 min and maximum lifting weight of the UAV was approximately 300 g. In the proposed UAV, the whole cell biosensor system includes a mechanical housing, a temperature regulating system, and a 300 µm height polydimethylsiloxane microfluidic channel for bacterial inoculation. The performance of the biosensor was evaluated by measuring the viability of *E. coli* bacteria in the biosensor, which was thus compared with standard bacterial culture techniques [87]. Thus, future sensing systems based on the conjugation of biosensors and drones are required for the environmental monitoring in remote locations due to their low-cost, compactness, and low power requirements.

Taking into account the biosensors discussed in this review paper, we could conclude that electrochemical and enzymatic biosensors are mostly used for environmental monitoring, as stated in Table 1, where the analytical characteristics of such biosensors are summarized. Particularly, biosensors with acetylcholinesterase have been the most attractive for pesticide determination due to their simplicity, specificity, cost-effectiveness, as well as due to the irreversible inhibition of acetylcholinesterase by such pesticides. Although enzymes have been used mostly as recognition elements in biosensors for pesticide detection since they are selective, their purification is costly and time-consuming, they have a poor thermal stability, and they are efficient only at an optimum pH and temperature. Thus, aptamers have also been an advantageous choice in biosensor recognition due to the possibility of designing their structure, denaturalizing and rehybridizing, and distinguishing targets with different functional groups, as well as due to their thermal stability and in vivo synthesis [1]. Immunosensors have also been reported for the detection of organic molecules such as toxins and endocrine disrupting chemicals. Although antibodies provided a high specificity with the corresponding antigen, some limitations such as poor regeneration and difficult immobilization on sensor substrates lead to the study of optimal conditions for antibody preparation and immobilization, which could be time-consuming and disadvantageous in sensor development. Other antibody characteristics such as number, orientation, and position on sensor surface could also influence the sensor response and optimization due to the loss of antibody activity.

The main limitation found for the recent environmental biosensors is based on the lack of application in real environmental samples since the majority of identified “environmental biosensors” has been applied to tap water samples or synthetic samples. Consequently, there are still few commercial biosensors for environmental monitoring, contrary to clinical applications, which should be due to the interdisciplinary context of fabrication as well as limitations on the in situ operation and on the analytical performance, mainly in reproducibility. The biosensors described in this review article were applied in real environmental conditions, that is, in real water samples (lake, river, seawater, soil and wastewater samples) or shellfish samples, making evident the recent effort to overcome this limitation.

## Figures and Tables

**Table 1:** Summary of recent biosensors for environmental monitoring.

Analyte/Pollutant Detected	Biosensor Type	Recognition Element	Electrode/Sensing Material	Reproducibility	Limit of Detection	Response Range	Recovery (%)	References
**Pesticides**								
Paraoxon	Electrochemical (amperometric)	Enzyme (AChE ^1^)	Gold SPE ^2^ and cysteamine SAM ^3^	5% (*n* = 4)	2 ppb (*^1^)	Up to 40 ppb	97 ± 5%	[4]
	Electrochemical (voltammetric)	Enzyme (butyrylcholinesterase)	SPE ^2^ with carbon black nanoparticles		5 µg L^−1^ (*^1^)	Up to 30 µg L^−1^	96 ± 2%	[13]
	Optical (colorimetric)	Enzyme (AChE ^1^ and ChO ^4^)	Iodine-starch		4.7 ppb (*^2^)	10–400 ppb	88–110%	[5]
	Electrochemical (amperometric)	Enzyme (AChE ^1^)	GCE ^5^ and gold nanorods	<6% (*n* = 6)	0.7 nM (*^1^)	1 nM–5 µM	96–98%	[2]
Methyl parathion	Electrochemical (impedimetric)	Enzyme (hydrolase)	SPE ^2^ with Fe_3_O_4_ and gold nanoparticles	7.8% (*n* = 6)	0.1 ng mL^−1^	0.5–1000 ng mL^−1^		[8]
	Electrochemical (amperometric)	Enzyme (AChE ^1^)	Graphite and macroalgae		1.5–1.8 ng mL^−1^ (*^1^)	0–1500 ng mL^−1^		[14]
	Electrochemical (impedimetric)	Enzyme (AChE ^1^)	Carbon paste electrode and reticulated spheres structures of NiCo_2_S_4_	5.3% (*n* = 6)	0.42 pg mL^−1^ (*^3^)	1.0 pg mL^−1^–10 ng mL^−1^		[15]
	Electrochemical	Enzyme (AChE ^1^)	Carbon paste electrode with chitosan, gold nanoparticles, and Nafion		5 fg mL^−1^	0.01 pg mL^−1^–10 ng mL^−1^		[16]
	Optical	*Sphingomonas* sp. cells	Microplate with silica nanoparticles and PEi ^6^ hybrid		0.01 ppm	0.1–1 ppm		[17]
Chlorpyrifos	Electrochemical (impedimetric)	Enzyme (tyrosinase)	SPCE ^7^ and IrO_x_ nanoparticles	<10% (*n* = 3)	3 nM	0.01–0.1 µM	90 ± 9.6%	[18]
	Electrochemical (voltammetric)	Enzyme (AChE ^1^)	Boron-doped diamond electrode with gold nanoparticles and carbon spheres	7.3% (*n* = 6)	0.13 pM (*^4^)	0.01 nM–0.1 µM	82.4–91.2%	[19]
	Electrochemical (voltammetric)	Aptamers (^#1^)	Carbon black and GO ^8^/Fe_3_O_4_	4.3% (*n* = 5)	94 pM (*^3^)	0.29 nM–0.29 mM	96–106%	[20]
	Electrochemical (amperometric)	Enzyme (AChE ^1^)	GCE ^5^ with NiO nanoparticles-carboxylic graphene-Nafion	6.5% (*n* = 6)	0.05 pM (*^2^)	0.1–10 nM	93.0–105.2%	[21]
Dichlorvos	Optical (fluorescence)	Enzyme (AChE ^1^ and ChO ^4^)	QD ^9^ and acetylcholine	2.2% (*n* = 6)	4.49 nM (*^1^)	4.49–6780 nM	97.1–100.9%	[22]
	Electrochemical (voltammetric)	Enzyme (AChE ^1^)	Platinum electrode with ZnO		12 pM (*^1^)		98.5–100.8%	[23]
	Electrochemical (impedimetric)	Enzyme (AChE ^1^)	Ionic liquids-gold nanoparticles porous carbon composite	6.5% (*n* = 5)	0.3 pM (*^1^)	0.45 pM–4.5 nM	80.8–93.1%	[24]
Acetamiprid	Optical (colorimetric)	Aptamers (^#2^)	Gold nanoparticles		5 nM (*^3^)	75 nM–7.5 µM		[25]
	Electrochemical (impedimetric)	Aptamers (^#3^)	Gold nanoparticles, MWCNT ^10^, and rGO ^11^ nanoribbons		17 fM (*^3^)	50 fM–10 µM	96.0–106.6%	[26]
	Electrochemical (impedimetric)	Aptamers (^#3^)	Silver nanoparticles and nitrogen-doped GO ^8^	6.9% (*n* = 5)	33 fM (*^3^)	0.1 pM–5 nM	98.8–106.5%	[27]
	Electrochemical (impedimetric)	Aptamers (^#3^)	Platinum nanoparticles		1 pM	10 pM–100 nM	86–109%	[28]
Atrazine	Electrochemical (voltammetric)	Antibodies (monoclonal)	Gold nanoparticles	2.7–9.2% (*n* = 3)	0.016 ng mL^−1^ (*^3^)	0.05–0.5 ng mL^−1^	95.5–119.9%	[29]
	Electrochemical (FET ^17^)	Antibodies (monoclonal)	SWCNT	1.86 ± 0.26%	0.01 ng mL^−1^	0.001–10 ng mL^−1^	87.3–108%	[30]
	Electrochemical (impedimetric)	Aptamers (^#4^)	Platinum nanoparticles		2.2 pg mL^−1^	22 pg mL^−1^–0.22 µg mL^−1^	79–113%	[28]
	Electrochemical (amperometric)	Phage/antibody (monoclonal) complex	Magnetic beads functionalized with protein G		0.2 pg mL^−1^	0.0001–0.001 pg mL^−1^	96–99%	[31]
Pirimicarb	Electrochemical (voltammetric)	Enzyme (laccase)	Carbon paste electrode with MWCNT ^10^	4.6% (*n* = 5)	43 µg L^−1^	0.24–2.7 mg L^−1^		[32]
	Electrochemical (amperometric)	Enzyme (AChE ^1^)	Prussian blue-MWCNT ^10^ SPE ^2^		53.2 ng L^−1^ (*^5^)	1 µg L^−1^–1 g L^−1^		[33]
Carbofuran	Electrochemical (voltammetric)	Enzyme (AChE ^1^)	IrO_x_-chitosan nanocomposite	5.4% (*n* = 5)	3.6 nM (*^2^)	5–90 nM		[34]
	Electrochemical (amperometric)	Enzyme (AChE ^1^)	GCE ^5^ with GO ^8^ and MWCNT^10^		136 pM	68–3672 pM	102.38 ± 2.05%	[35]
	Electrochemical (amperometric)	Enzyme (AChE ^1^)	GCE ^5^ with NiO nanoparticles-carboxylic graphene-Nafion composite	6.5% (*n* = 6)	0.5 pM (*^2^)	1 pM–0.1 nM	93.0–105.2%	[21]
Carbaryl	Electrochemical (impedimetric)	Enzyme (AChE ^1^)	Gold electrode with cysteamine SAM ^3^		32 nM	1–9 µM		[36]
	Electrochemical (impedimetric)	Enzyme (AChE ^1^)	Interdigitated array microelectrodes with chitosan	4.8%	3.87 nM	4.96–496 nM		[37]
	Electrochemical (amperometric)	Enzyme (AChE ^1^)	MWCNT ^10^ and GO ^8^ nanoribbons structure	7.3% (*n* = 4)	1.7 nM (*^3^)	5–5000 nM	95.5–96.8%	[38]
	Electrochemical (amperometric)	Enzyme (AChE ^1^)	Porous GCE ^5^ with GO ^8^ network		0.74 nM (*^3^)	1.49–30.3 nM	98.3–102.2%	[39]
**Pathogens**								
*Legionella pneumophila*	Optical (SPR ^12^)	Nucleic acids (^#5^)	Gold substrate with streptavidin-conjugated QD ^9^		10^4^ CFU mL^−1^	10^4^–10^8^ CFU mL^−1^		[40]
	Optical (SPR ^12^)	Antibody (polyclonal)	Gold substrate with protein A SAM ^3^		10^3^ CFU mL^−1^	10^3^–10^6^ CFU mL^−1^		[41]
	Electrochemical (amperometric)	Antibody (polyclonal)	SPCE ^7^ with Fe_3_O_4_@polydopamine complex	5.9% (*n* = 7)	10^4^ CFU mL^−1^	10^4^–10^8^ CFU mL^−1^		[42]
	Optical (SPR ^12^)	Antibody (polyclonal)	Gold gratings substrate		10 CFU mL^−1^			[43]
*Escherichia coli*	Optical (SPR ^12^)	Polymerizable form of histidine	Gold substrate		3.72 × 10^5^ CFU mL^−1^			[44]
	Piezoelectric (QCM ^13^)				1.54 × 10^6^ CFU mL^−1^			
	Electrochemical (capacitive)	Polymerizable form of histidine	Gold electrode		70 CFU mL^−1^	10^2^–10^7^ CFU mL^−1^	81–97%	[45]
	Optical (electrochemiluminescence)	Antibodies (polyclonal)	GCE ^5^ with polydopamine imprinted polymer and nitrogen-doped QD ^9^		8 CFU mL^−1^	10–10^7^ CFU mL^−1^		[46]
*Bacillus subtilis*	Electrochemical (amperometric)	Antibodies (polyclonal)	Gold electrode with SWCNT ^14^		10^2^ CFU mL^−1^	10^2^–10^10^ CFU mL^−1^		[47]
**Potentially Toxic Elements**								
Hg^2+^	Optical (evanescent-wave optical fibre)	Nucleic acids (^#6^)	Optical fibre platform		1.2 nM (*^2^)	0–1000 nM		[48]
	Optical (fluorescence)	DNA	MOF ^15^ (UiO-66-NH_2_)		17.6 nM	0.14 µM		[49]
	Electrochemical (voltammetric)	Nucleic acids (^#7^)	Gold substrate with vertically aligned SWCNT	3.4%	3 fM (*^3^)	10 fM–1 µM		[50]
	Optical (SERS ^16^)	Nucleic acids (^#8^)	SWCNT ^11^ and CoFe_3_O_4_@Ag substrate	<4% (*n* = 15)	0.84 pM (*^3^)	1 pM–100 nM	90.50–116.7%	[51]
Pb^2+^	Optical (fluorescence)	DNAzymes (^#9^)	Carboxylated magnetic beads		5 nM (*^3^)	0–50 nM	96.1–101%	[52]
	Optical (fluorescence)	DNAzyme (^#10^)	Graphene QD ^9^ and gold nanoparticles		16.7 nM	50 nM–4 µM		[53]
	Optical (fluorescence)	Aptamers (^#11^)	Micro-spin column	<5% (*n* = 6)	61 nM (*^3^)	100–1000 nM	95.2–109.3%	[54]
**Toxins**								
Brevetoxin-2	Electrochemical (impedimetric)	Aptamers (^#12^)	Gold electrodes with cysteamine SAM ^3^		106 pg mL^−1^	0.01–2000 ng mL^−1^	102–110%	[55]
	Electrochemical (voltammetric)	Cardiomyocyte cells	Microelectrode array with platinum nanoparticles		1.55 ng mL^−1^	5.6 ng mL^−1^–1.4 µg mL^−1^		[56]
Saxitoxin	Electrochemical (voltammetric)	Cardiomyocyte cells	Microelectrode array with platinum nanoparticles		0.35 ng mL^−1^	5.6 ng mL^−1^–1.4 µg mL^−1^		[56]
	Optical (interferometry)	Aptamers			0.5 ng mL^−1^	10–2000 ng mL^−1^	101.4–107.3%	[57]
Microcystin	Electrochemical (impedimetric)	Antibodies (monoclonal)	Graphene	6.9%	50 pg mL^−1^	0.05–20 ng mL^−1^		[58]
	Electrochemical (voltammetric)	Antibodies (monoclonal)	Gold electrodes with MoS_2_ and gold nanorods		5 pg mL^−1^ (*^3^)	0.01–20 ng mL^−1^	98.3–102.1%	[59]
	Electrochemical (voltammetric)	Enzyme (protein phosphate 1)	SPE ^2^		0.93 ng mL^−1^ (*^1^)	0.93–40.32 ng mL^−1^		[60]
Okadaic acid	Optical (SPR ^12^)	Antibodies	Gold electrode with carboxymethylated surface		0.36 ng mL^−1^			[61]
	Electrochemical (FET ^17^)	Antibodies (monoclonal)	Graphene	0.54–2.19% (*n* = 5)	0.05 ng mL^−1^	0.05–300 ng mL^−1^	98.2–100.7%	[62]
	Optical (fluorescence)	Antibodies (monoclonal)	Carboxylic acid modified magnetic beads and CdTe QD ^9^		0.05 ng mL^−1^	0.2–20 ng mL^−1^		[63]
Domoic acid	Optical (SPR ^12^)	Antibodies	Gold electrode with carboxymethylated surface		1.66 ng mL^−1^			[61]
	Electrochemical (FET ^17^)	Antibodies (monoclonal)	SWCNT ^14^	0.52–1.43% (*n* = 5)	10 ng mL^−1^	10–500 ng mL^−1^	92.3–100.3%	[64]
	Optical (SPR ^12^)	Antibodies	Glass side chip with gold surface		0.1 ng mL^−1^	0.1–2 ng mL^−1^		[65]
**Endocrine Disrupting Chemicals**								
Bisphenol A	Optical (fluorescence)	Aptamers	Gold nanoparticles		0.1 ng mL^−1^	1–10000 ng mL^−1^		[66]
	Optical (evanescent-wave optical fibre)	Aptamers (^#13^)	Optical fibre surface		0.45 ng mL^−1^ (*^2^)	460 pg mL^−1^–22.8 ng mL^−1^	91–110%	[67]
	Optical (fluorescence)	Aptamers (^#14^)	Molybdenum carbide nanotubes		0.23 ng mL^−1^	0–91.3 ng mL^−1^		[68]
Nonylphenol	Electrochemical (FET ^17^)	Antibodies (monoclonal)	SWCNT ^14^	0.56 ± 0.08% (*n* = 5)	5 ng mL^−1^	5–500 ng mL^−1^	97.8–104.6%	[69]
17β-estradiol	Photo-electrochemical	Aptamers (^#15^)	CdSe nanoparticles and TiO_2_ nanotubes	6.33% (*n* = 5)	33 fM	0–80 pM	90.0–102.8%	[70]
	Electrochemical (voltammetric)	Antibodies	Gold electrode with MPA ^18^ SAM ^3^		2.25 pg mL^−1^	2.25–2250 pg mL^−1^		[71]
	Electrochemical (capacitive)	Antibodies	Gold electrode with MUA ^19^ SAM ^3^		1 pg mL^−1^ (*^3^)	1–200 pg mL^−1^	97.96–102%	[72]

Notes: ^1^ AChE: acetylcholinesterase, ^2^ SPE: screen printed electrode, ^3^ SAM: self-assembled monolayer, ^4^ ChO: choline oxidase, ^5^ GCE: glassy carbon electrode, ^6^ PEi: polyethyleneimine, ^7^ SPCE: screen printed carbon electrode, ^8^ GO: graphene oxide, ^9^ QD: quantum dots, ^10^ MWCNT: multi-walled carbon nanotubes, ^11^ rGO: reduced graphene oxide, ^12^ SPR: surface plasmon resonance, ^13^ QCM: quartz crystal microbalance, ^14^ SWCNT: single-walled carbon nanotubes, ^15^ MOF: metal organic framework, ^16^ SERS: surface enhancement Raman spectrum, ^17^ FET: field effect transistor, ^18^ MPA: 3-mercaptopropionic acid, ^19^ MUA: 11-mercaptoundecanoic acid; *^1^ LOD is calculated at 10% of inhibition of the total enzymatic activity, *^2^ LOD is 3 s/k with s the standard deviation and k the slope of calibration plot, *^3^ LOD is three times the signal-to-noise ratio, *^4^ LOD is calculated at 15% of inhibition of the total enzymatic activity, *^5^ LOD is calculated at 5% of inhibition of the total enzymatic activity; Oligonucleotides sequences: ^#1^ 5′-CCTGCCACGCTCCGCAAGCTTAGGGTTACGCCTGCAGCGATTCTTGATCGCGCTGCTGGTAATCCTTCTTTAAGCTTGGCACCCGCATCGT-3′, ^#2^ 5′-CTGACACCATATTATGAAGA-3′, ^#3^ 5′-(SH)-(CH_2_)_6_-TGTAATTTGTCTGCAGCGGTTCTTGATCGCTGACACCATATTATGAAGA-3′, ^#4^ 5′-(SH)-(CH_2_)_6_-TACTGTTTGCACTGGCGGATTTAGCCAGTCAGTG-3′; ^#5^ 5′-CTCTGTATCGGCCATTGTAGC-3′, ^#6^ 5′-NH_2_-(CH_2_)_6_-GTACAAACAA-3′; ^#7^ 5′-NH_2_-(CH_2_)_6_-GCTAAGCCATAGATCAATGCGCGGGACTGTCTTT-3′, ^#8^ 5′-SH-(CH_2_)_6_-TCATGTTTGTTTGTTGGCCCCCCTTCTTTCTTA-3′; ^#9^ 5′-TAGTCTACTCTCTGAAGTAGCGCCGCCGTAGTGTAC-3′, ^#10^ 5′-/3ThioMC3-D/CGATAACTCACTATrAGGAAGAGATG-3′, ^#11^ 5′-GGAGGCTCTCGGGACGACGTCGTCCCGATGCTGCAATCGTAAGAAT-3′, ^#12^ 5′-ATACCAGCTTATTCAATTAGATAGTAAGTGCAATCT-3′, ^#13^ 5′-Cy5.5-CCGGTGGGTGGTCAGGTGGGATAGCGTTCCGCGTATGGCCCAGCGCATCACGGGTTCGCACCA-3′, ^#14^ 5′-CCGGTGGGTGGTCAGGTGGGATAGCGTTCCGCGTATGGCCCAGCGCATCACGGGTTCGCACCA-3′, ^#15^ 5′-GCTTCCAGCTTATTGAATTACACGCAGAGGGTAGCGGCTCTGCGCATTCAATTGCTGCGCGCTGAAGCGCGGAAGC-3′.
